# P35 A 4 year evaluation of antibiotic prophylaxis in robotic and robotic-assisted prostatectomy with or without lymph node dissection at St Vincent’s University Hospital

**DOI:** 10.1093/jacamr/dlaf230.042

**Published:** 2025-12-04

**Authors:** Clarice Egan, Grace Rothwell-Kelly, Sinead McNicholas

**Affiliations:** Department of Clinical Microbiology, St Vincent’s University Hospital, Dublin, Ireland; Department of Clinical Microbiology, St Vincent’s University Hospital, Dublin, Ireland; Department of Clinical Microbiology, St Vincent’s University Hospital, Dublin, Ireland

## Abstract

**Background:**

Robotic-assisted laparoscopic prostatectomy (RALP) is the preferred surgical intervention for localized prostate cancer due to its minimally invasive nature, reduced blood loss and lower complication rates. It is routine practice in St Vincent’s University Hospital (SVUH) since its introduction in 2017. RALP is generally considered a clean-contaminated procedure, warranting surgical antimicrobial prophylaxis (SAP) to reduce infection risk. There are currently no specific local guidelines for SAP in this context, however, in line with international guidelines, SAP for urological procedures in SVUH is usually a single dose. The SVUH Enhanced Recovery Protocol for RALP recommends three doses of antibiotics, the equivalent of 24 h. European Association of Urology Guidelines 2025 found insufficient evidence to support or oppose SAP in RALP. CDC 2017 Surgical Site Infection Prevention Guideline recommends SAP be timed to ensure bactericidal concentrations are present when the incision is made and advises against additional antimicrobial doses once the surgical incision is closed in clean-contaminated procedures. American Urological Association (AUA) guidelines recommend SAP in all cases of RALP with a single antimicrobial dose.^6^

**Objectives:**

To assess current SAP practices in RALP at SVUH, their alignment with microbiological data and opportunities for optimizing antibiotic use.

**Methods:**

(i) Retrospective review of RALP procedures at SVUH between 2021 and 2024. (ii) Data collected included intra-operative antibiotic administration, urine culture results within 6 months pre-operatively and post-operative blood culture results. (iii) SAP appropriateness was determined by comparing administered antibiotics to susceptibility profiles of isolated Gram-negative organisms. (iv) Statistical analysis using Microsoft Excel.

**Results:**

A total of 467 RALP procedures were performed, 11% included pelvic lymph node dissection. All were elective, median patient age was 66 years and median operation time was 189 min. Prophylactic antibiotic data was available for 454 patients. Co-amoxiclav (89%) was most commonly used, followed by gentamicin, ciprofloxacin, cefuroxime, teicoplanin and meropenem. Only 42 patients (9.0%) had urine cultures within six months pre-operatively, eight of which had Gram-negative growth that could guide prophylaxis. In 98% (446/454) of cases prophylaxis was empirical, 99% (442/446) of which were deemed appropriate (4/446 patients received teicoplanin only in the absence of Gram-negative cover or known MRSA colonization). In 2% (8/454) of cases prophylaxis could be tailored, 75% (6/8) of which were deemed appropriate (2/8 patients received antibiotics ineffective against known resistant organisms). Blood cultures were taken during the same admission in 15 (3%) patients, with no Gram-negative bacteraemia detected.

**Conclusions:**

This evaluation confirms routine SAP use in RALP at SVUH, predominantly with co-amoxiclav. Although only intra-operative antibiotic administration was captured, standard practice is three doses, then stop. In line with CDC recommendations and other Irish local Hospital guidelines, we advocate a shift toward single-dose prophylaxis tailored to surgical duration, blood loss and local resistance data. Updating SVUH’s antimicrobial guidelines and Enhanced Recovery Protocol for RALP is essential to reduce unnecessary antibiotic use and mitigate antimicrobial resistance. Patients should have surveillance urine samples taken pre-operatively to guide prophylaxis.

